# The Quantification of Acceleration Events in Elite Team Sport: a Systematic Review

**DOI:** 10.1186/s40798-021-00332-8

**Published:** 2021-06-30

**Authors:** Robert I. M. Delves, Robert J. Aughey, Kevin Ball, Grant M. Duthie

**Affiliations:** 1 Institute for Health & Sport, Victoria University, Melbourne, VIC 3011 Australia; 2 School of Behavioural and Health Sciences, Australian Catholic University, Strathfield, NSW Australia

**Keywords:** Acceleration, Data filtering, Activity profile, Deceleration, Wearable technology, Athlete tracking, Microtechnology, Athlete loads

## Abstract

**Background:**

Wearable tracking devices are commonly utilised to quantify the external acceleration load of team sport athletes during training and competition. The ability to accelerate is an important attribute for athletes in many team sports. However, there are many different acceleration metrics that exist in team sport research. This review aimed to provide researchers and practitioners with a clear reporting framework on acceleration variables by outlining the different metrics and calculation processes that have been adopted to quantify acceleration loads in team sport research.

**Methods:**

A systematic review of three electronic databases (CINAHL, MEDLINE, SPORTDiscus), was performed to identify peer-reviewed studies that published external acceleration load in elite team sports during training and/or competition. Articles published between January 2010 and April 2020 were identified using Boolean search phrases in relation to team sports (population), acceleration/deceleration (comparators), and competition and/or training (outcome). The included studies were required to present external acceleration and/or deceleration load (of any magnitude) from able-bodied athletes (mean age ≥ 18 years) via wearable technologies.

**Results:**

A total of 124 research articles qualified for inclusion. In total, 113/124 studies utilised GPS/GNSS technology to outline the external acceleration load of athletes. Count-based metrics of acceleration were predominant of all metrics in this review (72%). There was a lack of information surrounding the calculation process of acceleration with 13% of studies specifying the filter used in the processing of athlete data, whilst 32% outlined the minimum effort duration (MED). Markers of GPS/GNSS data quality, including horizontal dilution of precision (HDOP) and the average number of satellites connected, were outlined in 24% and 27% of studies respectively.

**Conclusions:**

Team sport research has predominantly quantified external acceleration load in training and competition with count-based metrics. Despite the influence of data filtering processes and MEDs upon acceleration, this information is largely omitted from team sport research. Future research that outlines acceleration load should present filtering processes, MEDs, HDOP, and the number of connected satellites. For GPS/GNSS systems, satellite planning tools should document evidence of available satellites for data collection to analyse tracking device performance. The development of a consistent acceleration filtering method should be established to promote consistency in the research of external athlete acceleration loads.

## Key Points

- Acceleration in team sport research has largely been quantified via the use of count-based metrics.
- There is a lack of information surrounding the processing of acceleration data in team sport research. Very few studies in this review quantified the filtering processes used to calculate acceleration and the minimum effort duration for these events. For satellite-based tracking systems, inconsistency seen in GPS/GNSS device reporting on horizontal dilution of precision and satellite number information has hindered the ability to objectively evaluate athlete acceleration and deceleration datasets.
- Future research should attempt to develop a common acceleration filtering/processing method to allow for appropriate comparison in load between studies and between tracking manufacturers. A common process would help to alleviate concerns of technology-driven variations in athlete acceleration data.

## Background

Through the continued development of athlete wearable technology, team sport practitioners have increasingly elected to monitor their athlete’s external load during training and competition with player-tracking devices [1]. Technologies, such as the Global Positioning System (GPS) and optical-based systems, are established player-tracking methods, whilst progressions have been made in the development of local positioning systems (LPS) and access to the Global Navigation Satellite System (GNSS). Regardless of the technology implemented, the aforementioned tracking systems allow for the relatively unobtrusive and objective collection of a player’s locomotion during training and match-play, with information obtained on athlete distances and speeds [1, 2]. Tracking information allows for the creation of activity profiles for respective sports, which details the different load placed upon athletes and positions played within that sport [1, 3]. For performance staff, an activity profile enables specific prescription of athlete training programs and rehabilitation processes that are centred towards preparing the athlete for the rigours of competition load [1].

The ability to change speed and direction through acceleration and deceleration are important attributes for successful performance in many team sports [4–7]. Subsequently, team sport research has produced a wide variety of metrics to assess acceleration in training and competition [7, 8]. Given the stochastic nature of team sport movement, the assessment of acceleration is imperative in depicting the overall loads of competition [7]. For example, team sport athletes across the football codes of rugby league, rugby union, association (soccer) and Australian football represent average match speeds that would be considered low intensity at approximately 80 to 140 m min^− 1^ (1.3–2.3 m s^− 1^) [7]. However, the aforementioned sports can see peak intensities up to 170 to 210 m min^− 1^ during a 1-min moving average epoch and have been shown to further increase to intensities up to 380 m min^− 1^ with smaller moving average window lengths (e.g., 5 s) [6, 9–12]. The wide range in intensities from match averages to competition peaks indicates that the ability to change velocity (acceleration) is important to performance. In invasion/combat sports such as rugby league, where general play is contested in tight confines, acceleration load is highest compared to other football codes, indicating the ability to rapidly change velocity is important to successful performance in this code [6, 9–11]. Similarly, in American football, where players are also actively trying to gain or negate yardage, skill players such as wide receivers, defensive backs and line-backers accumulate substantial counts of high accelerations (> 3.5 m s^− 2^) per game (range 26–38 counts per game) [13].

Whilst being able to perform accelerations is important to successful athletic performance, quantifying accelerations is also important to practitioners for athlete load management [8]. Accelerations incorporate a significant portion of the total overall external load during team sport training and competition [8, 14–17]. However, the magnitude of acceleration efforts can provide different sources of load experienced by the athlete. For example, accelerations (positive velocity) will place a greater metabolic cost on the body compared to deceleration events, as accelerations require greater energy to fuel the change in velocity [4, 14, 15, 18]. Deceleration events however differ from accelerations with respect to the mechanically demanding, eccentric loads placed upon the body when braking (particularly at higher intensities). Athlete braking (decelerating) is dampened by soft-tissue structures which attempt to attenuate the force of each deceleration effort [8, 14–17, 19]. In team sport athletes, an increased count of high-intensity accelerations is associated with neuromuscular fatigue and muscle damage (marked by increased creatine kinase) post competition [7, 8, 14, 20]. Therefore, it is important that acceleration and deceleration can be appropriately quantified and monitored during training and competition to ensure athletes are adequately prepared for this load [7, 9].

For team sport practitioners and researchers however, the existing research on acceleration and how acceleration load in competition and training is quantified, has varied greatly between studies [7, 8]. Currently, there are a multitude of different methods in which to quantify accelerations in team sport research [21]. Specifically, acceleration in applied team sports has been quantified via threshold based counts, time or distance spent in certain thresholds (e.g., > 3.5 m s^− 2^ threshold for “high-intensity accelerations”) or more recently, by combining all absolute acceleration data (regardless of intensity) and averaging over a defined time period [1, 7, 20–23].

Regardless of the metric chosen to quantify acceleration, the measurement of acceleration is subject to the device quality and filtering settings of the tracking system. In GPS technology, there have been continual improvements in device capabilities, with 10-Hz devices being deemed most valid and reliable for measuring acceleration [3, 7, 22, 24]. Varley et al. [22] determined that 10-Hz devices could, at worst, detect an acceleration had occurred, but otherwise possessed acceptable validity for accelerations at various starting velocities in straight running (CV 3.6–5.9%). However, deceleration at a starting velocity between 5 and 8 m s^− 1^ had greater variability (CV 11.3%) which was attributed to the rapid change in speed during deceleration compared to acceleration [7, 22, 24].

To analyse the quality of positional data in GPS/GNSS devices, the horizontal dilution of precision (HDOP) and the average number of connected satellites are extracted [1, 25]. For GPS/GNSS devices, HDOP and the number of satellites provide an indication of the quality of device connection and signal strength [2, 25]. However, despite the importance of HDOP and the number of satellite information, the reporting of these metrics has been inconsistent in team sport research [1]. With the development of online GNSS planning tools providing evidence of the number of available satellites for a given period, researchers and practitioners should endeavour to compare the satellite tracking information from their devices to website-based tools outlining satellite availability. Extracting satellite quality information can then aid in assessing the overall data quality of metrics surrounding acceleration events. Given the importance of device signal quality on athlete positioning data, the HDOP and the number of connected satellites are significant variables that need to be reported upon in athlete-tracking research. In practice, the publishing of HDOP and satellite data then aids practitioners to determine what data they should include and exclude in their athlete load monitoring systems, including acceleration metrics. For example, HDOP values greater than one or satellite numbers less than 10 may be grounds for data exclusion in daily monitoring processes.

The processing or calculation of an acceleration event may also influence the measurement of athlete acceleration [1]. It is believed that despite the similarities in device hardware between manufacturers, the filtering and minimum effort durations in the calculation of acceleration/deceleration largely differ between devices, potentially creating technology-driven differences in acceleration/deceleration-based research [1, 26, 27]. Despite the previously stated need for greater consistency in the reporting of wearable device specifications and processes, there are still large inconsistencies in reporting of acceleration in team sport research.

With the ongoing development of athlete-tracking systems as a measure of external athlete output and the approval to implement these devices during competition, there is an increasing prevalence of the technology in team sport research [1, 8]. Additionally, with the extensive number of studies that have outlined activity profiles of respective sports during training and competition, numerous systematic reviews have been published [8, 28–30]. However, there is currently no systematic review that has outlined the different metrics and the calculation of the metrics used to quantify accelerations in team sport research. The systematic review from Harper et al. [8] outlined and compared high and very high-intensity accelerations in competitive team sports but this study was dependent upon cut-off thresholds, which limited the overall scope of the study. The introduction of metrics such as absolute acceleration prompted this review to include all acceleration events/metrics regardless of the magnitude, as ultimately all acceleration and deceleration events carry a physiological cost [7]. With the inevitable further developments in player-tracking technologies (e.g., optical systems) and the importance of accelerations in team sport activity profiles, it is pertinent to review and appraise the metrics that have been used to quantify acceleration/deceleration. Therefore, the primary aim of this systematic review is to outline and compare the different methods that have been adopted to quantify acceleration and deceleration events in team sport research. A secondary aim was to identify the processing methods used by researchers in calculating acceleration/deceleration by way of data filtering methods and minimum effort durations.

## Methods

### Study Design

The current systematic review was undertaken in accordance with the Preferred items for Systematic Reviews and Meta-Analyses (PRISMA) statement on the transparent reporting of systematic reviews [31].

### Search Strategy

Three electronic databases (CINAHL, Medline, and SPORTDiscus) were systematically reviewed in May 2020 by the lead author to identify articles that investigated the quantification of acceleration and/or deceleration as a metric in the load monitoring of team sport athletes in either training or competitive environments. Peer-reviewed research articles published in the English language between January 1, 2010, and April 2020 were reviewed for selection into the study. The search terms devised for this review were constructed using the PICO framework, where population (team sport/team sport athletes), interest (quantification of Acceleration/Deceleration metrics) and context (in competition or training) were accounted for. Search terms and exclusion criteria (Table 1) relating to team sport athletes and the quantification of acceleration and deceleration in competition or training were then identified (Table 2). Boolean operators “OR” and “AND” were used in the final search to combine all search terms together (Table 2).

**Table 1:** Search inclusion and exclusion criteria

Study inclusion/exclusion criteria	
Inclusion criteria	Exclusion criteria
Original research articles	Systematic Reviews, Reviews, letters to the editors, non-peer reviewed articles, editorial, books, periodicals, surveys, opinion pieces, conference abstracts
Team-based sports	Outdoor court games (tennis, volleyball) water-based, ice-based and sand-based sports.
Participants with a mean age ≥ 18 years	Research with the mean age of athletes below the age of ≤ 18 years.
Elite-level, able-bodied, participants playing at the elite domestic competition for their respective team sport or international representation above U/18 competition	Sub-elite-level, amateur and novice athletes or athletes not playing within the top tier of their respective domestic league/competitions. Athletes with a physical or mental disability. Referees & Officials
Official team activities: including competition/game/match observations and training sessions (e.g., small sided games, match simulations, individual training drills)	Validation or reliability studies on wearable technologies using athletes in an experimental setting
GPS/GNSS-based trackers (sampling ≥ 5 Hz) Optical/LPS-based Camera Systems	Accelerometers
Acceleration or deceleration events measured during designated team activities of any magnitude and measured in any available metric (e.g., counts, metres, time spent, average acceleration, acceleration load) that is not combined with any separate metric (e.g., metabolic power)	Combined metrics (metabolic power, repeat high-intensity efforts, PlayerLoad)
Research available in English (full text)	Research articles that are not published in English or cannot be accessed in English.

**Table 2:** Search terms and keywords used in each database. Searches 1, 2, 3 and 4 were combined with “AND”

Key search terms	Related search terms
1. Acceleration/Deceleration	accelerat* OR decelerat* OR metabolic power OR metabolic load OR energetic cost
2. Athlete tracking System	global positioning system* OR GPS OR global navigation satellite system* OR GNSS OR local positioning system* OR LPS OR microtechnology OR microsensor* OR tracking system* OR athlete tracking system OR notational analysis OR camera-based tracking OR optical tracking system
3.Team sport	team sport* OR team-sport* OR intermittent sport OR professional team sport OR elite sport OR elite team sport OR australian rules football OR australian rules OR australian football OR australian football league OR AFL OR australian football team OR australian rules football team OR australian football club OR australian rules football club OR soccer OR soccer player OR soccer team OR football OR footballer OR football player OR football team OR field hockey OR field hockey athlete OR field hockey player OR rugby league OR rugby OR rugby league player OR rugby league team OR rugby football OR rugby league competition OR rugby union OR rugby union player OR rugby union competition OR rugby union club OR rugby sevens OR rugby sevens competition OR lacrosse OR lacrosse competition OR american football OR american football player OR national collegiate athletic association OR NCAA OR gaelic football OR gaelic football player OR hurling OR hurling player OR cricket OR netball OR basketball
4.Training/competition	movement demands OR movement pattern OR external load OR external demands OR physical workload OR physical demand* OR activity demand* OR activity profile OR activit* profile* OR match profile OR match demand* OR match play OR match-play OR match intensit* OR game load* OR game intensit* OR competit* demand* OR training OR training demands OR practice OR small sided games OR match simulation OR game simulation

### Screening Strategy and Study Selection

Upon execution of the search, all returned studies were collated and exported into a reference manager (EndNote X9, Thomson Reuters, Philadelphia, PA, USA) for further review. The initial review process incorporated three stages to identify qualifying articles. Firstly, all duplicate articles were identified and removed from the reference manager. Secondly, studies were scanned via their abstracts and keywords to establish relevance. If studies were deemed to be irrelevant at this juncture, they were excluded. If doubt remained after inspection of the abstract as to the relevance of the study, it would advance to the next stage for further scrutiny. The final stage consisted of reviewing the full-text documents of each study and excluding articles that were subject to the exclusion criteria (Table 1). If doubt remained as to the eligibility of respective studies following this process, the authors resolved the process through deliberation. If an article was identified through this process or identified in any other way other than the initial search it would be subject to the same review process to determine qualification.

### Data Extraction

All relevant search data were extracted into a custom-made Microsoft Excel spreadsheet by the lead author. The extracted data from each eligible study included athlete population (sport, competition, age, height, weight), athlete-tracking system used (e.g., GPS, LPS or camera-based) and the associated properties (e.g., unit sample rate, HDOP, number of satellites), acceleration metrics measured (e.g., counts, distance, or average acceleration), the filtering/processing method used to quantify the acceleration and any relevant acceleration findings. All acceleration events, regardless of the magnitude were included into the analysis. There were no exclusion criteria based on the velocity threshold of the acceleration event. Similarly, all organised team activities (training and competition) were eligible for inclusion into the study. Studies that only presented information on athlete-tracking device reliability or validity in an experimental setting were excluded from analysis. Additionally, given the recent guidance on the reporting of GPS/GNSS device properties in research and similar systematic review publications, all available GPS/GNSS device information was extracted from each relevant study [1, 8]. Specifically, the characteristics observed included HDOP, number of satellites connected during activity, device sample rate, device model and device manufacturer.

## Results

### Search Results

**Fig. 1:**
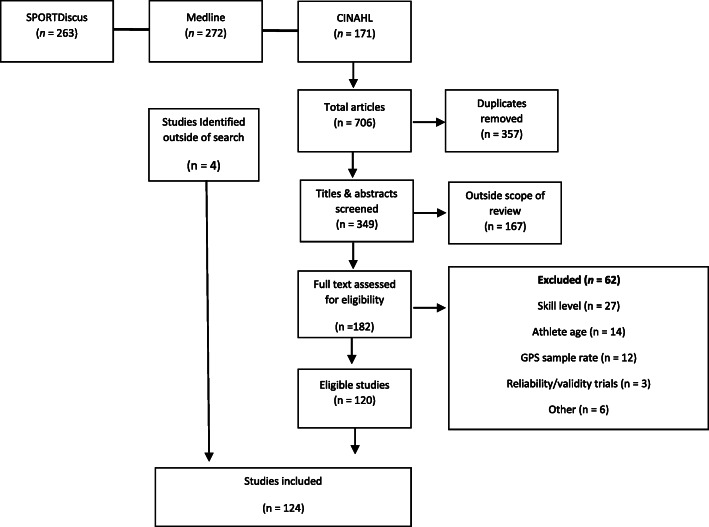
Systematic review inclusion process for qualification into the review

The combined search of three databases returned 706 studies (SPORTSDiscus = 263, Medline = 272, CINAHL = 171) for analysis. All 706 studies were exported into a reference manager where 357 articles were removed as being duplicates. This resulted in the screening of 349 titles and abstracts. Of these titles and abstracts, 167 articles were deemed well outside the scope of the review and were subsequently removed. In total, 182 full-text articles were reviewed and assessed relative to the parameters of the inclusive criteria. Upon review of all full-text articles, 62 were excluded based on athlete skill level (n = 27), athlete age (n = 14), GPS device sample rate (n = 12), inappropriate study type (n = 3) and other exclusions (including accelerometer derived acceleration and the use of combined metrics such as metabolic power) (n = 6). A total of 120 studies remained at the completion of this process. Additionally, four studies were identified and included outside of the database search via the review process for this research. Therefore, 124 studies were included. Figure 1 identifies the classification of studies and pathway of eligibility into the study.

### Study Characteristics

The accepted studies in this review outlined acceleration load during an organised, elite team sport activity. This was measured through various player-tracking technologies, including GPS/GNSS, local positioning systems or optical-based tracking systems. The results of this review are focused on how acceleration was quantified in these studies and the metrics used to present the external acceleration load. The characteristics of each of the included studies are summarised in Table 3.

**Table 3:** Tracking technology and acceleration/deceleration characteristics of each included study

Study	Team sport	Device	Manufacturer	Model	Sample rate (Hz)	HDOP	No. of satellites	Acc/dec	Filter	Calculation interval/MED	Threshold (m s^− 2^)	Acc/dec metric	Calculation of metric
Akenhead et al. [32]	Soccer	GPS	Catapult Sports	MinimaxX S4	10 Hz	0.9 ± 0.1	12 ± 1	Acc Dec	Smoothing Filter 0.5 s	0.5 s	Low: 1–2 Moderate: 2–3 High: > 3 Total: > 1	Distance (m)	Distance attained in respective threshold band. Acc/dec also pooled at 1 and 3 m s^−2^
Akenhead et al. [33]	Soccer	GPS	Catapult Sports	MinimaxX	10 Hz	0.8 ± 0.1	13 ± 1	Acc Dec	N/S	N/S	Low: 1–2 Moderate: 2–3 High: > 3 Total: > 1	Acc & Dec Distance (m)	Threshold-based sum of acc/dec distances
Akiyama et al. [34]	Lacrosse	GPS	Polar Electro	Polar Team Pro	10 Hz	N/S	N/S	Acc Dec	N/S	N/S	Low: 0–1.99 Moderate: 2.0–3.99 High: > 4	Counts (n)	Efforts in respective threshold band
Altavilla et al. [35]	Soccer	GPS	K-Sport	N/S	10 Hz	N/S	N/S	Acc Dec	N/S	N/S	High: > 2	Distance (m)	Distance attained in respective threshold band
Aughey [36]	Australian Football	GPS	Catapult Sports	MinimaxX Team Sport 2.0	5 Hz	N/S	N/S	Acc	N/S	0.4 s	Maximal: > 2.78	Counts (n) Counts per minute (n/min^−2^)	At least two consecutive efforts at same rate of change in velocity (0.4 s) respective threshold band
Aughey [37]	Australian Football	GPS	Catapult Sports	MinimaxX Team Sport 2.0	5 Hz	N/S	N/S	Acc	N/S	0.4 s	Maximal: > 2.78	Counts (n) Counts per minute (n/min^−2^)	At least two consecutive efforts at same rate of change in velocity (0.4 s) respective threshold. Efforts with respect to activity time
Aughey [38]	Australian Football	GPS	Catapult Sports	MinimaxX Team Sport 2.0	5 Hz	1.5 ± 0.9	7.5 ± 1.2	Acc	N/S	N/S	Maximal: > 2.78	Counts (n) Counts per minute (n/min^−2^)	Efforts with respect to activity time
Aughey [39]	Australian Football	GPS	Catapult Sports	MinimaxX Team Sport 2.0	5 Hz	N/S	N/S	Acc	N/S	0.4 s	Maximal: > 2.78	Counts (n) Counts per minute (n/min^−2^)	At least two consecutive efforts at same rate of change in velocity (0.4 s) respective threshold band. Efforts with respect to activity time
Bauer et al. [40]	Australian Football	GPS	Catapult Sports	MinimaxX v4.0	10 Hz	1.8 ± 0.4	N/S	Acc	N/S	N/S	Low: 0–2.77 Hard: ≥ 2.78	Counts (n) Distance (m)	Efforts in respective threshold band. Distance attained in respective threshold band.
Bayliff et al. [41]	American Football	GPS	Catapult Sports	Optimeye S5	10 Hz	N/S	N/S	Acc Dec	N/S	N/S	Band 1: 0–1 Band 2: 1–2 Band 3: 2–3 Band 4: 3–10	Distance (m)	Metres attained in respective threshold band
Blair et al. [42]	Rugby Sevens	GPS	GPSports	SPI Pro 10	10 Hz	N/S	N/S	Acc Dec	N/S	N/S	Low: 1.5–2.5 High: > 2.5-3.6	Counts (n)	Efforts in respective threshold band
Bowen et al. [43]	Soccer	GPS Optical	STATSports ChyronHego	Viper 2 TRACAB	10 Hz	N/S	N/S	Acc Dec	N/S	0.5 s	All: > 0.5	Counts (n)	Efforts in respective threshold band lasting at least 0.5 s and > 0.5 m s^−2^
Bradley et al. [44]	Soccer	Optical	ProZone Sports	ProZone Version 3.0	N/A	N/A	N/A	Acc	N/S	N/S	Medium: > 2.5-4 High: > 4	Counts (n)	Efforts in respective threshold band
Brooks et al. [45]	Netball	LPS	Catapult Sports	Catapult T6 ClearSky	10 Hz	N/A	N/A	Acc Dec	N/S	0.2 s	Z1: 0–2 Z2: 2–3.5 Z3: 3.5–6 Z4: 6–10	Acceleration Density: (Average Acc/Dec) (m s^−2^) Acceleration Density Index: (avg Acc/Dec per 10 m; m s^− 2^) Total Acceleration Load: (total Acc/Dec; m s^− 2^) Distance (m)	Average acc values across the specified period Average acc performed per 10 m of distance (Acc Load/Distance) Sum of acc values across the analysed period (acc values were calculated at 0.2 s intervals) Distance attained in respective threshold
Campos-Vazquez et al. [46]	Soccer	GPS	Catapult Sports	MinimaxX S4	10 Hz	N/S	N/S	Acc Dec	N/S	N/S	Moderate: 2–3 High: > 3	Distance per hour (m h^−1^)	Distance attained in respective threshold
Chesher et al. [47]	Field Hockey	GPS	Catapult Sports	MinimaxX S4	10 Hz	0.88 ± 0.03	11 ± 0.59	Dec	N/S	N/S	Low: − 3–−5.99 Medium: -6–− 8.99 High: − 9–− 11.99 Very high: <− 12	Counts (n) Average Deceleration (m s^−2^)	Efforts in respective threshold band. Mean Dec in each competitive match
Clemente et al. [48]	Soccer	GPS	JOHAN Sports	N/S	10 Hz	N/S	N/S	Acc Dec	N/S	N/S	High: > 3	Counts (n)	Efforts in respective threshold band
Couderc et al. [49]	Rugby Sevens	GPS	Digital Simulation	Sensor Everywhere	8 Hz	N/S	N/S	Acc	Butterworth low pass 2^nd^ order Cut-off frequency: 1 Hz Double phase lag filter	0.5 s	High: > 2.5	Counts (n)	Efforts in respective threshold band
Coutts et al. [50]	Australian Football	GPS	Catapult Sports	MinimaxX Team Sport 2.5	10 Hz	N/S	N/S	Acc Dec	N/S	0.2 s	> 2.78	Counts (n)	Two consecutive samples exceeding 2.78 m s^−2^
Cummins et al. [51]	Rugby League	GPS	GPSports	SPI Pro X	15 Hz^a^	N/S	N/S	Acc Dec	Butterworth 4^th^ order Cut-off frequency: 1 Hz	N/S	Moderate: < 1.12 High: 1.13–2.78 Very high: > 2.78	Counts per minute (n/min^−2^)	Efforts in respective threshold band with respect to activity time
Cummins et al. [52]	Rugby League	GPS	Catapult Sports	Optimeye S5	10 Hz	N/S	N/S	Acc Dec	N/S	N/S	All: > 1.5	Counts (n)	Efforts in respective threshold band
Cummins et al. [53]	Rugby League	GPS	GPSports	SPI Pro X	15 Hz^a^	N/S	N/S	Acc Dec	Butterworth 4^th^ Order Cut-off frequency: 1 Hz	N/S	Moderate: < 1.12 High: 1.13–2.78 Very high: > 2.78	Counts (n)	Efforts in respective threshold band
Cunningham et al. [54]	Rugby Union	GPS	STATSports	Viper	10 Hz	N/S	N/S	Acc Dec	N/S	N/S	Moderate: 2–3 High: 3–4 Severe: > 4	Counts (n)	Efforts in respective threshold band
Cunningham et al. [55]	Rugby Union	GPS	STATSports	Viper	10 Hz	N/S	4 Best Satellites	Acc Dec	N/S	N/S	Moderate: 2–3 High intensity: 3–4 Severe: > 4	Counts (n)	Efforts in respective threshold band
Dalen et al. [56]	Soccer	Radio Freq. Tracking	ZXY Sport Tracking	RadioEye Sensors	20 Hz	N/A	N/A	Acc	N/S	0.5 s	All: > 2	Counts per minute (n/min^−2^)	Efforts lasting for at 0.5 s in respective threshold band. Efforts in respective threshold band with respect to activity time
Dalen et al. [57]	Soccer	Radio Freq. Tracking	ZXY Sport Tracking	RadioEye Sensors	20 Hz	N/A	N/A	Acc Dec	N/S	0.5 s	All: > 2	Counts (n) Distance (m)	Efforts lasting for at 0.5 s in respective threshold band
de Hoyo et al. [17]	Soccer	GPS	GPSports	SPI Elite	10 Hz	N/S	N/S	Acc Dec	N/S	N/S	Moderate: 2–3 High: > 3	Counts (n)	Efforts in respective threshold band
Delaney et al. [7]	Rugby League	GPS	GPSports	SPI HPU	5 Hz	N/S	N/S	Acc Dec	N/S	N/S	Low: 1 Moderate: 2 High: > 3	Counts (n) Time (s) Distance (m) Average Acc (m s^−2^) Average Dec (m s^− 2^) Average Acc/Dec (m s^− 2^)	Efforts, time and/or distance in respective threshold band. Absolute values of acc averaged over given analysis period. Absolute values of dec averaged over given analysis period. Absolute values of acc/dec averaged over given analysis period
Delaney et al. [9]	Rugby League	GPS	GPSports	SPI HPU	15 Hz^a^	1.1 ± 0.1	8.3 ± 1.4	Acc Dec	Butterworth 4^th^ Order Cut-off frequency: 1 Hz	N/S	N/A	Average Acc (m s^−2^) / min	Absolute values of acc/dec averaged over given analysis period
Delaney et al. [10]	Australian Football	GPS	Catapult Sports	Optimeye S5	10 Hz	N/S	N/S	Acc Dec	N/S	N/S	N/A	Average Acc (m s^−2^) / min	Absolute values of acc/dec averaged over given analysis period
Delaney et al. [11]	Rugby Union	GPS	GPSports	SPI HPU	15 Hz^a^	N/S	N/S	Acc Dec	N/S	N/S	N/A	Average Acc (m s^−2^) / min	Absolute values of acc/dec averaged over given analysis period
Delaney et al. [6]	Soccer	GPS	Catapult Sports	Optimeye S5	10 Hz	0.86 ± 0.28	10.6 ± 1.7	Acc Dec	N/S	N/S	N/A	Average Acc (m s^−2^) / min	Absolute values of acc/dec averaged over given analysis period
Delves et al. [58]	Field Hockey	GPS	Catapult Sports	Optimeye X4 MinimaxX S4	10 Hz	N/S	N/S	Acc Dec	N/S	N/S	N/A	Average Acc (m s^−2^) / min Average Acc (m s^−2^)	Absolute values of acc/dec averaged over given analysis period
Dempsey et al. [59]	Rugby League	GPS	GPSports	SPI Pro X	10 Hz	N/S	N/S	Acc Dec	N/S	N/S	High: > 3.0	Counts (n) Counts per minute (n/min^−2^)	Efforts in respective threshold band. Efforts calculated in absolute terms with respect to activity time and threshold
Dubois et al. [60]	Rugby Union	GPS	GPSports	SPI HPU	15 Hz^a^	N/S	N/S	Acc Dec	N/S	N/S	All: > 2.5	Counts (n)	Efforts in respective threshold band
Duthie et al. [61]	Field Hockey	GPS	Catapult Sports	Optimeye X4	10 Hz	N/S	N/S	Acc Dec	N/S	N/S	N/A	Average Acc (m s^−2^) / min	Absolute values of acc/dec averaged over given analysis period
Figueiredo et al. [62]	Soccer	GPS	STATSports	Viper Pod	10 Hz	N/S	N/S	Acc Dec	N/S	N/S	N/S	Counts (n)	Efforts in respective threshold band
Furlan et al. [63]	Rugby Sevens	GPS	GPSports	SPI HPU	5 Hz	N/S	N/S	Acc Dec	Butterworth 4^th^ Order Cut-off frequency: 1 Hz	N/S	Moderate: 2–3 High: .1–4 Very high: > 4	Counts per minute (n/min^−2^)	Acc/Dec counts derived from filtered 15 Hz data. Efforts in respective threshold band with respect to activity time
Gabbett [64]	Rugby League	GPS	Catapult Sports	MinimaxX	5 Hz	N/S	N/S	Acc	N/S	N/S	Mild: 0.55–1.11 Moderate: 1.12–2.78 Maximal: > 2.79	Counts (n)	Efforts in respective threshold band
Gabbett [65]	Field Hockey	GPS	Catapult Sports	MinimaxX	5 Hz	N/S	N/S	Acc	N/S	2 s	High: > 0.5	Counts (n)	Efforts in respective threshold band lasting at least 2 s
Gabbett [66]	Rugby League	GPS	Catapult Sports	MinimaxX Team Sport 2.5	5 Hz	N/S	N/S	Acc	N/S	N/S	Maximal: > 2.79	Counts (n) Counts per minute (n/min^−2^)	Efforts in respective threshold band. Efforts in respective threshold band with respect to activity time
Gabbett et al. [67]	Rugby League	GPS	Catapult Sports	MinimaxX	5 Hz	N/S	N/S	Acc	N/S	N/S	Mild: 0.55–1.11 Moderate: 1.12–2.78 Maximal: > 2.79	Distance (m)	Distance in respective threshold band
Gabbett & Ullah [68]	Rugby League	GPS	Catapult Sports	MinimaxX	5 Hz	N/S	N/S	Acc	N/S	N/S	Mild: 0.55–1.11 Moderate: 1.12–2.78 Maximal: > 2.79	Distance (m)	Distance in respective threshold band
Garvican et al. [69]	Soccer	GPS	Catapult Sports	MinimaxX Team Sport 4.0	10 Hz	N/S	N/S	Acc	N/S	N/S	Maximal: > 2.78	Counts (n) Counts per minute (n/min^−2^)	Efforts in respective threshold band. Efforts in respective threshold band with respect to activity time
Gaudino et al. [70]	Soccer	GPS	GPSports	SPI Pro X	15 Hz^a^	N/S	Range: 8-11 Satellites	Acc Dec	N/S	1 s	Moderate: 2–3 High: > 3	Counts (n) Maximum Acc/Dec (m s^−2^)	Efforts in respective threshold band lasting for at least 1 s. Maximum acc & dec effort in analysed period.
Gaudino et al. [71]	Soccer	GPS	STATSports	Viper	10 Hz	N/S	N/S	Acc Dec	N/S	0.5 s	Total: > 3	Counts (n) Counts per minute (n/min^−2^)	Efforts in respective threshold band lasting for at least 0.5 s and of magnitude > 0.5 m s^− 2^
Hauer et al. [72]	Lacrosse	GPS	Polar Electro	Polar Team Pro	10 Hz	N/S	N/S	Acc Dec	N/S	N/S	Z1: 0–1.0 Z2: 1.0–2.0 Z3: 2.0–3.0 Z4: > 3.0	Counts (n)	Efforts in respective threshold band
Higham et al. [73]	Rugby Sevens	GPS	Catapult Sports	MinimaxX Team Sport 2.5	5 Hz	N/S	N/S	Acc Dec	N/S	0.4 s	Moderate: 2 – 4 High: > 4	Counts per minute (n/min^− 2^)	Efforts in respective threshold band with respect to activity time
Higham et al. [74]	Rugby Sevens	GPS	GPSports	SPI Pro X	15 Hz^a^	N/S	N/S	Acc Dec	N/S	1 s	Total: > 1	Counts per minute (n/min^−2^ )	Efforts in respective threshold band with respect to activity time lasting at least 1 s.
Hoppe et al. [75]	Soccer	GPS	Catapult Sports	MinimaxX S4	10 Hz	1.1 ± 0.1	11.8 ± 0.5	Acc Dec	Butterworth 2 Passes Cut-off: 1 Hz	N/S	High:> 3	Time (s)	Time spent in respective threshold band
Ihsan et al. [76]	Field Hockey	GPS	Catapult Sports	MinimaxX Team Sport 2.5	5 Hz	N/S	N/S	Acc Dec	N/S	N/S	High: > 2	Counts (n)	Efforts in respective threshold band
Ingebrigtsen et al. [77]	Soccer	Radio Tracking	ZXY SportTracking	ZXY Sport Chip	40 Hz	N/A	N/A	Acc	N/S	0.5 s	Total: > 2	Counts (n)	1) The start of Acc is marked by the Acc reaching the minimum limit (1 m s). 2) Acc has to reach 2 m s. 3) Acc must remain above the 2 m s for at least 0.5 s. 4) The duration of the Acc lasts until it passes the minimum Acc limit (1 m s)
Jackson et al. [78]	Field Hockey	GPS	Catapult Sports	MinimaxX S4 Optimeye S5	10 Hz	MinimaxX: (0.89 [0.04]) Optimeye S5: (0.67 [0.05])	N/S	Acc Dec	Smoothing Filter	0.2 s–Calc 0.6 s– Minimum effort duration	Total: > 1.46 Maximum count per athlete	Counts (n) Maximum Acc/Dec (m s^−2^)	Efforts in respective threshold band. Max Acc/Dec recorded
Jaspers et al. [79]	Soccer	GPS	Catapult Sports	Optimeye S5	10 Hz	N/S	N/S	Acc Dec	N/S	N/S	Z1: 0–1 Z2: 1–2 Z3: 2–3.5 Z4: > 3.5	Counts (n) Distance (m)	Efforts in respective threshold band. Distance attained in respective threshold band
Jaspers et al. [80]	Soccer	GPS	Catapult Sports	Optimeye S5 MinimaxX S4	10 Hz	< 1.5	≥8 satellites	Acc Dec	Smoothing Filter 0.2 s	0.4 s	Total: > 1	Counts (n)	Efforts in respective threshold band
Johnston et al. [81]	Rugby League	GPS	STATSports	Apex	10 Hz	0.76 ± 0.25	17.7 ± 1.9	Acc Dec	N/S	N/S	N/A	Average Acc (m s^−2^) / min	Absolute values of acc/dec averaged over given analysis period
Johnston et al. [82]	Australian Football Rugby League	GPS	AF: Catapult Sports RL: STATSports	AF: Optimeye S5 RL: Apex	10 Hz	AFL: 0.69 ± 0.09 NRL: 0.76 ± 0.25	AFL: 10.5 ± 0.65 NRL: 17.7 ± 1.90	Acc Dec	N/S	N/S	N/A	Average Acc (m s^−2^) / min	Absolute values of acc/dec averaged over given analysis period
Johnston et al. [83]	Rugby League	GPS	Catapult Sports	Optimeye S5	10 Hz	N/S	N/S	Acc Dec	N/S	N/S	N/A	Average Acc (m s^−2^) / min	Absolute values of acc/dec averaged over given analysis period
Johnston et al. [84]	Australian Football	GPS	Catapult Sports	MinimaxX S3 MinimaxX S4	S3: 5 Hz S4: 10 Hz	1.0 ± 0.3	12.2 ± 0.7	Acc Dec	N/S	N/S	Low: 0.65–1.46 Moderate: 1.47–2.77 High: > 2.78	Counts per minute (n/min^−2^) Distance per minute (m/min) Time (%)	Efforts calculated in absolute terms with respect to activity time and threshold. Distance in respective threshold band with respect to activity time and threshold. Time spent as a percentage in respective threshold band
Johnston et al. [85]	Australian Football	GPS	Catapult Sports	MinimaxX S3 MinimaxX S4	5 Hz 10 Hz	1.0 ± 0.2	12.1 ± 0.7	Acc Dec	N/S	N/S	Low: 0.65–1.46 Moderate: 1.47–2.77 High: > 2.78	Counts per minute (n/min^−2^) Distance per minute (m/min^−2^) Time (%. min^−2^)	Efforts in respective threshold band with respect to activity time and threshold. Distance attained in respective threshold band with respect to activity time and threshold. Percentage time spent in respective threshold band with respect to activity time
Johnston et al. [86]	Australian Football	GPS	Catapult Sports	MinimaxX S3 MinimaxX S4	5 Hz 10 Hz	1.0 ± 0.1	12.2 ± 0.6	Acc Dec	N/S	N/S	Low: 0.65–1.46 Moderate: 1.47–2.77 High: > 2.78	Counts per minute (n/min^−2^) Distance per minute (m/min^−2^) Time (%. min^−2^)	Efforts in respective threshold band with respect to activity time. Distance attained in respective threshold band with respect to activity time. Percentage time spent in respective threshold band with respect to activity time
Jones et al. [87]	Rugby Union	GPS	Catapult Sports	MinimaxX V4	10 Hz	N/S	N/S	Acc Dec	N/S	N/S	Low: 1–2 Moderate: 2–3 High: > 3	Distance (m)	Metres attained in respective threshold band
Kempton & Coutts [88]	Rugby League Nines	GPS	GPSports	SPI Pro X	15 Hz^a^	N/S	N/S	Acc Dec	N/S	N/S	Total: > 2.78	Counts (n) Counts per minute (n/min^−2^)	Efforts in respective threshold band. Efforts calculated in absolute terms with respect to activity time and threshold
Kempton et al. [89]	Rugby League	GPS	GPSports	SPI Pro X	15 Hz^a^	N/S	N/S	Acc Dec	N/S	N/S	Total: > 2.78	Counts (n) Counts per minute (n/min^−2^)	Efforts in respective threshold band. Efforts calculated in absolute terms with respect to activity time and threshold
Kempton et al. [90]	Rugby League	GPS	GPSports	SPI Pro	5 Hz	N/S	9.1 ± 1.4	Acc Dec	N/S	0.4 s	Total: > 2.78	Counts (n)	Two consecutive samples exceeding 2.78 m s^−2^
Lacome et al. [91]	Rugby Union	PC-based tracking	Sport Universal Process	Amisco Pro	10 Hz Velocity	N/A	N/A	Acc	Butterworth low pass 2^nd^ order cut-off frequency: 1 Hz Double phase-lag filter	0.5 s	Z1: 1–2 Z2: 2–3 Z3: > 3	Mean acceleration (m s^−2^)	Values of acc averaged over given analysis period. Distribution of acc values over given analysis period with respect to thresholds
Malone et al. [92]	Soccer	GPS	Catapult Sports	Optimeye G5	10 Hz	N/S	N/S	Acc Dec	N/S	N/S	High: > 3	Counts (n)	Efforts in respective threshold band
Mara et al. [93]	Soccer	GPS	GPSports	N/S	15 Hz^a^	N/S	5-8 Satellites	Acc	N/S	N/S	Efforts: > 2	Time (s) Distance (m) Max distance (m) Max acceleration (m s^−2^) Repeat acceleration	Average time spent in acc in analysed period. Average distance accumulated in analysed period. Average max distance accumulated in analysed period. Max acc effort in analysed period Acc efforts performed with < 21 s separation
Mara et al. [94]	Soccer	GPS	GPSports	SPI HPU	15 Hz^a^	N/S	N/S	Acc Dec	N/S	N/S	High: > 2	Counts (n)	Efforts in respective threshold band
Marrier et al. [95]	Rugby Sevens	GPS	Digital Simulation	Sensor Everywhere V2	16 Hz	< 2	7 [1]	Acc	N/S	0.5 s	All: > 2.5	Counts (n)	Efforts in respective threshold band lasting for at least 0.5 s
Martin-Garcia et al. [96]	Soccer	GPS	STATSports	Viper	10 Hz	N/S	N/S	Acc Dec	N/S	N/S	High: > 3	Counts (n)	Efforts in respective threshold band
Martin-Garcia et al. [97]	Soccer	GPS	STATSports	Viper	10 Hz	N/S	N/S	Acc Dec	N/S	0.5 s	High: > 3	Counts (n)	Efforts in threshold band lasting for at least 0.5 s and of magnitude > 0.5 m s^−2^
Martin-Garcia et al. [98]	Soccer	GPS	STATSports	Viper	10 Hz	N/S	N/S	Acc Dec	N/S	N/S	High: > 3	Counts (n)	Efforts in respective threshold band
Modric et al. [99]	Soccer	GPS	Catapult Sports	Optimeye S5 Optimeye X4	10 Hz	N/S	N/S	Acc Dec	N/S	N/S	Total events: > 0.5 High: > 3	Counts (n)	Efforts in respective threshold band
Montgomery & Maloney [100]	3 × 3 Basketball	GPS	Catapult Sports	Optimeye S5	10 Hz	N/S	N/S	Acc Dec	N/S	N/S	Low: < 2.5 Medium: 2.5–3.5 High: > 3.5	Intensity (m s^−2^)	Average intensity in respective threshold band.
Morencos et al. [101]	Field Hockey	GPS	GPSports	SPI Elite	10 Hz	N/S	10.6 ± 1.2	Acc Dec	N/S	N/S	Low: 1–1.99 Moderate: 2.0–2.99 High: > 3	Counts (n) Counts per minute (n/min^−2^)	Efforts in respective threshold band. Efforts calculated in absolute terms with respect to activity time and threshold
Morencos et al. [102]	Field Hockey	GPS	GPSports	SPI Elite	10 Hz	N/S	N/S	Acc Dec	N/S	N/S	Low: 1.0–1.9 Moderate: 2.0–2.9 High: > 3.0	Counts (n) Counts per minute (n/min^−2^)	Efforts in respective threshold band. Efforts in respective threshold band with respect to activity time
Murray & Varley [103]	Rugby Sevens	GPS	Catapult Sports	MinimaxX S4	10 Hz	N/S	11.3 ± 1.4	Acc	N/S	0.4 s	Maximal: > 2.78	Counts (n) Counts per minute (n/min^−2^)	Efforts in respective threshold band lasting at least 0.4 s. Efforts calculated in absolute terms with respect to activity time and threshold lasting at least 0.4 s
Newans et al. [104]	Soccer	GPS	Catapult Sports	Optimeye S5 Optimeye X4	10 Hz	N/S	N/S	Acc Dec	N/S	0.5 s	Moderate: 1–2 High: > 2	Time (s) Ratio of Dec: Acc	Time spent in each respective threshold lasting at least 0.5 s. Duration of Dec (High) and Dec (Mod) divided by total Acc time (High + Mod) in each period. Determined a moderate and high Dec:Acc ratio
Owen et al. [105]	Soccer	GPS	STATSports	Viper Pod	10 Hz	N/S	N/S	Acc Dec	N/S	N/S	Total: > 3.3	Counts (n)	Efforts in respective threshold band
Owen et al. [106]	Rugby Union	GPS	GPSports	SPI HPU	15 Hz^a^	N/S	N/S	Acc Dec	N/S	N/S	Light: 1–1.99 Moderate: 2.0–2.99 Heavy: 3–5.99	Counts (n)	Efforts in respective threshold band
Oxendale et al. [107]	Rugby League	GPS	Catapult Sports	MinimaxX Team Sport 2.5	10 Hz	N/S	N/S	Acc Dec	N/S	N/S	Maximal: > 2.79	Counts (n)	Efforts in respective threshold band
Palmer et al. [108]	Ultimate Frisbee	GPS	Catapult Sports	Optimeye X4	10 Hz	0.90 ± 0.10	13.7 ± 0.5	Acc	Proprietary Filter	0.6 s	Total: > 1.5	Counts (n) Counts per minute (n/min^−2^)	Efforts in respective threshold band lasting for at least 0.6 s and with respect to time
Passos Ramos et al. [109]	Soccer	GPS	Catapult Sports	MinimaxX Team S5	10 Hz	N/S	N/S	Acc Dec	N/S	N/S	1: − 1–1 2: 1–2.5 3: > 2.5	Counts (n) Counts per minute (n/min^−2^)	Efforts in respective threshold band and with respect to time
Passos Ramos et al. [110]	Soccer	GPS	Catapult Sports	MinimaxX Team S5	10 Hz	0.75 ± 0.3	12.4 ± 0.5	Acc Dec	Exponential Filter (Derived from GPS Software)	0.5 s	Total: > 1	Counts (n)	Efforts in respective threshold band
Passos Ramos et al. [111]	Soccer	GPS	Catapult Sports	MinimaxX Team S5	10 Hz	0.75 ± 0.3	15.5 ± 0.5	Acc Dec	N/S	N/S	Total: > 2	Counts (n)	Efforts in respective threshold band
Peeters et al. [112]	Rugby Sevens	GPS	Digital Simulation	Sensor Everywhere	16 Hz	1.35 ± 0.34	8 ± 1	Acc	N/S	N/S	Total: > 2.5	Counts (n) Counts per minute (n/min^−2^)	Efforts in respective threshold band. Efforts calculated in absolute terms with respect to activity time
Polgaze et al. [113]	Field Hockey	GPS	Catapult Sports	MinimaxX S4	10 Hz	1.00 ± 0.07	11.6 ± 0.5	Acc	Proprietary Filter	0.6 s	Low: < 2.0 High: > 2.0	Counts (n) Counts per minute (n/min^−2^) Time (s) Distance (m)	Eligible Acc was determined once a participant changed speed by 2 m s for a minimum within 0.6 s. Efforts in respective threshold band. Efforts calculated in absolute terms with respect to activity time and threshold Time spent in respective threshold band. Distance attained in respective threshold band
Pollard et al. [114]	Rugby Union	GPS	STATSports	Viper	10 Hz	N/S	N/S	Acc	N/S	N/S	Total: > 3	Counts per minute (n/min^−2^)	Efforts in respective threshold band. Efforts in respective threshold band with respect to activity time
Polley et al. [115]	Lacrosse	GPS	Catapult Sports	MinimaxX S4	10 Hz	N/S	N/S	Acc Dec	N/S	N/S	Low: 0–1.11 Moderate: 1.11–2.78 High: ≥ 2.78	Counts per minute (n/min^−2^)	Efforts in respective threshold band with respect to activity time
Portillo et al. [116]	Rugby Sevens	GPS	GPSports	SPI Pro X	15 Hz^a^	N/S	N/S	Acc	N/S	N/S	Z1: > 1.5 Z2: > 2.0 Z3: > 2.5 Z4: > 2.75	Counts (n)	Efforts in respective threshold band
Rennie et al. [117]	Australian Football	GPS	Catapult Sports	Optimeye S5	10 Hz	1.1 ± 0.1	18.2 ± 1.1	Acc Dec	N/S	0.2 s Two Samples	Efforts: > 2.78	Counts (n)	Two consecutive samples in respective threshold band
Romero-Moraleda et al. [118]	Field Hockey	GPS	GPSports	SPI Elite	10 Hz	N/S	N/S	Acc Dec	N/S	N/S	Low: 1–1.9 Moderate: 2–2.9 High: > 3	Counts per minute (n/min^−2^)	Efforts in respective threshold band with respect to activity time
Russell et al. [119]	Soccer	GPS	STATSports	Viper	10 Hz	N/S	N/S	Acc Dec	N/S	N/S	Total: > 0.5 High: > 3	Counts (n)	Efforts in respective threshold band
Russell et al. [120]	Soccer	GPS	STATSports	Viper	10 Hz	N/S	N/S	Acc Dec	N/S	0.5 s	Total: > 0.5 High: > 3	Counts (n)	Efforts in respective threshold band
Sangnier et al. [121]	Soccer	GPS	K-Sport	K-GPS	10 Hz	N/S	N/S	Acc Dec	N/S	0.4 s (over 3 s threshold)	Distance: > 2 Counts: > 3	Counts per minute (n/min^−2^) Distance per min (m/min)	Efforts > 0.4 s (over 3 m s^−2^ threshold). Distance in threshold band with respect to activity time
Silva et al. [122]	Soccer	GPS	STATSports	Viper	10 Hz	N/S	N/S	Acc Dec	N/S	0.5 s	Z1: > 2 Z2: > 2.5 Z3: > 3	Counts (n) Counts per minute (n/min^−2^)	Efforts in respective threshold band lasting at least 0.5 s of magnitude > 0.5 m s^−2^
Smpokos et al. [123]	Soccer	GPS	STATSports	Viper Pod 2	10 Hz	N/S	N/S	Acc Dec	N/S	0.5 s	Total: > 2	Counts (n) and counts per minute (n/min^−2^)	Efforts in respective threshold band lasting at least 0.5 s of magnitude > 0.5 m s^−2^
Smpokos et al. [124]	Soccer	GPS	STATSports	Viper Pod 2	10 Hz	N/S	N/S	Acc Dec	N/S	0.5 s	Total: > 2	Counts (n) and counts per minute (n/min^−2^)	Efforts in respective threshold band lasting at least 0.5 s of magnitude > 0.5 m s^−2^
Stevens et al. [125]	Soccer	LPS	Inmotio	Inmotio LPS	24 Hz	N/A	N/A	Acc	Weighted Gaussian Average	N/S	> 2	Distance (m)	Distance in respective threshold band
Stevens et al. [126]	Soccer	LPS	Inmotio LPS	Inmotio LPS	31 Hz	N/A	N/A	Acc Dec	Weighted Gaussian Average	0.5 s	Medium: > 1.5 High: > 3	Counts (n)	Efforts in respective threshold band
Suarez-Arrones et al. [127]	Rugby Sevens	GPS	GPSports	SPI Pro X	15 Hz^a^	N/S	N/S	Acc Dec	N/S	1 s	Maximal: 2.78–4 Extremely high: > 4	Counts (n)	1-s at > 2.78 m s^−2^ or above
Suarez-Arrones et al. [128]	Rugby Union	GPS	GPSports	SPI Pro X	15 Hz^a^	N/S	N/S	Acc	N/S	N/S	Maximal: > 2.78	Counts (n)	Efforts in respective threshold band
Sullivan et al. [129]	Australian Football	GPS	Catapult Sports	MinimaxX Team Sport 2.5	10 Hz	1.25 ± 0.19	N/S	Acc	N/S	N/S	One Threshold: 0–4	Counts per minute (n/min^−2^)	Efforts with respect to activity time
Sullivan et al. [130]	Australian Football	GPS	Catapult Sports	MinimaxX Team Sport 2.5	10 Hz	1.25 ± 0.19	N/S	Acc	N/S	N/S	One Threshold: 0–4	Counts per minute (n/min^−2^)	Efforts with respect to activity time
Sweeting et al. [131]	Netball	Radio Tracking System	WASP	WASP Node	10 Hz	N/A	N/A	Acc	Kalman Filter	N/S	N/A	Intensity-based clusters (m s^−2^)	Acceleration calculated from velocity data.
Tee et al. [132]	Rugby Union	GPS	GPSports	SPI Pro	10 Hz	N/S	N/S	Acc	N/S	N/S	Efforts: > 2.75	Minutes per Accel (n/min)	Efforts with respect to activity time
Tee et al. [133]	Rugby Union	GPS	GPSports	SPI Pro	5 Hz	N/S	N/S	Acc	N/S	1 s	Maximal: > 2.75	Minutes per Accel (n/min)	Efforts in respective threshold band with respect to activity time
Tee et al. [134]	Rugby Union	GPS	GPSports	SPI Pro	5 Hz	N/S	N/S	Acc	N/S	N/S	Total: > 2.75	Minutes per Accel (n/min)	Efforts in respective threshold band with respect to activity time
Thornton et al. [135]	Rugby League	GPS	GPSports	SPI HPU	15 Hz^a^	N/S	N/S	Acc Dec	N/S	N/S	N/A	Acc/Dec Load (AU)	Average absolute value of all acc/dec data relative to a defined period. Absolute value multiplied by defined duration to convert to load metric
Varley & Aughey [136]	Soccer	GPS	GPSports	SPI Pro	5 Hz	N/S	8 ± 1	Acc	N/S	N/S	Maximal: > 2.78	Counts (n)	Efforts in respective threshold band
Varley et al. [137]	Soccer Rugby League Australian Football	GPS	AF & RL: Catapult Sports Soccer: GPSports	AF & RL: MinimaxX Team Sport 2.5 Soccer: SPI Pro	5 Hz	N/S	N/S	Acc	N/S	N/S	Maximal: > 2.78	Counts (n) Counts per minute (n/min^−2^)	Efforts in respective threshold band. Efforts in respective threshold band with respect to activity time
Vazquez-Guerrero et al. [138]	Basketball	LPS	Realtrack Systems	WIMU Pro	20 Hz	N/A	N/A	Acc Dec	N/S	N/S	Total Acc: All counts High intensity: > 2	Peak acceleration (m s^−2^) Counts (n) Counts per minute (n/min^−2^)	Highest acc value obtained during analysed period. Efforts in respective threshold band. Efforts in respective threshold band with respect to activity time
Vescovi & Frayne [139]	Field Hockey	GPS	GPSports	SPI Pro	5 Hz	Values < 4	8–12 Satellites connected during collection	Acc Dec	N/S	N/S	All events	Counts (n)	Efforts in respective threshold band
Vigh-Larsen et al. [140]	Soccer	Radio Tracking System	Chryon-Hego	ZXY Tracking System	20 Hz	N/A	N/A	Acc Dec	N/S	0.5 s	Total: > 2	Counts (n) Counts per minute (n/min^−2^)	Efforts lasting at least 0.5 s and reaching at least 1 m s^−2^ Efforts lasting for at least 0.5 s and reaching at least 1 m s^−2^.
Wehbe et al. [141]	Soccer	GPS	GPSports	SPI Pro	5 Hz	N/S	N/S	Acc Dec	N/S	0.5 s	Medium: 2.5–4 High: > 4	Counts (n)	Efforts in respective threshold band lasting at least 0.5 s
Wellman et al. [142]	American Football	GPS	Catapult Sports	MinimaxX S5	10 Hz	N/S	N/S	Acc Dec	N/S	N/S	Low: 0–1.0 Medium: 1.1–2.0 High: 2.1–3.0 Maximal: > 3.0	Distance (m)	Distance attained in respective threshold band
Wellman et al. [143]	American Football	GPS	Catapult Sports	Optimeye S5	10 Hz	N/S	N/S	Acc Dec	N/S	N/S	Low: 0–1.0 Medium: 1.1–2.0 High: 2.1–3 Maximal: >− 3	Distance (m)	Distance attained in respective threshold band
Wellman et al. [13]	American Football	GPS	GPSports	SPI HPU	15 Hz^a^	N/S	N/S	Acc Dec	N/S	N/S	Moderate: 1.5–2.5 High: 2.6–3.5 Maximal: > 3.5	Counts (n)	Efforts in respective threshold band
White & MacFarlane [144]	Field Hockey	GPS	Catapult Sports	MinimaxX	5 Hz	Scotland Analysis: 1.3 ± 0.4 Ukraine Analysis: 1.0 ± 0.4	Scotland Analysis: 12.3 ± 1.0 Ukraine Analysis: 10.3 ± 1.2	Acc	N/S	N/S	High: > 2	Counts (n)	Efforts in respective threshold band
White & MacFarlane [145]	Field Hockey	GPS	Catapult Sports	MinimaxX	5 Hz	Scotland Analysis: 1.3 ± 0.4 Ukraine analysis: 1.0 ± 0.4	Scotland Analysis: 12.3 ± 1.0 Ukraine Analysis: 10.3 ± 1.2	Acc Dec	N/S	1 s	High: > 2	Counts (n)	Efforts in respective threshold band lasting for at least 1 s
White & MacFarlane [146]	Field Hockey	GPS	Catapult Sports	MinimaxX	5 Hz	0.99 ± 0.2	11.2 ± 1.3	Acc	N/S	> 1 s	High intensity: > 3	Counts (n)	Efforts in respective threshold band lasting at least 0.5 s
Yamamoto et al. [147]	Rugby Union	GPS	GPSports	SPI Pro X	5 Hz	N/S	N/S	Acc	N/S	N/S	AZ1: 1.5–2.0 AZ2: 2–2.5 AZ3: > 2.5	Counts (n)	Efforts in respective threshold band
Young et al. [148]	Hurling	GPS	STATSports	Viper Pod	10 Hz	N/S	N/S	Acc Dec	N/S	N/S	Total: > 2	Counts (n)	Efforts in respective threshold band

^a^15-Hz device interpolated from 5 Hz

### Team Sport Characteristics

The team sport characteristics of each of the 124 studies are featured in Table 4. Of the 124 articles, research from Soccer provided the greatest contribution of studies to the review (33.9%), followed by Rugby League (14.2%), Australian Football (11.8%) and Field Hockey (11.0%). Athlete sex was mixed in each sport contribution, with the exception of Australian and American Football, Basketball, Hurling, Rugby League, Rugby League Nines and Ultimate Frisbee.

**Table 4:** Characteristics of studies

Sport	Study count	% sport contribution to review	Study athlete sex		Athlete level	Reference
			% Male	% Female		
3 × 3 Basketball	1	0.8	50	50	Elite, Junior International	[100]
American Football	4	3.1	100	0	Elite Collegiate	[13, 41, 142, 143]
Australian Football	15	11.8	100	0	Elite	[10, 36–40, 50, 82, 84–86, 117, 129, 130, 137]
Basketball	1	0.8	100	0	Elite	[138]
Field Hockey	14	11.0	66	33	Elite, Elite Collegiate	[47, 58, 61, 65, 76, 78, 101, 102, 113, 118, 139, 144–146]
Hurling	1	0.8	100	0	Elite	[148]
Lacrosse	3	2.4	66	33	Elite	[34, 72, 115]
Netball	2	1.6	0	100	Elite	[45, 131]
Rugby League	18	14.2	100	0	Elite	[7, 9, 51–53, 59, 64, 66–68, 81–83, 89, 90, 107, 135, 137]
Rugby League Nines	1	0.8	100	0	Elite	[88]
Rugby Sevens	10	7.9	90	10	Elite	[42, 49, 63, 74, 95, 103, 112, 116, 127]
Rugby Union	13	10.2	92	8	Elite, Junior International	[11, 54, 55, 60, 87, 91, 106, 114, 128, 132–134, 147]
Soccer	43	33.9	88	12	Elite, Junior International	[6, 17, 32, 33, 35, 43, 44, 46, 48, 56, 57, 62, 69–71, 75, 77, 79, 80, 92–94, 96–99, 104, 105, 109–111, 119–126, 136, 137, 140, 141]
Ultimate Frisbee	1	0.8	0	100	Junior International	[108]
Total	**127**	**100**	**75**	**25**		

### Tracking Device Characteristics

The wearable technology type, as well as respective manufacturers and devices, are outlined in Table 5. Global Positioning System/GNSS-based studies were assessed on two data quality metrics. HDOP (mean ± SD) and the number of satellites (mean ± SD) in connection with the GPS device during athlete tracking were observed in this review. Of the 113 eligible GPS/GNSS studies, 23.9% (27/113 studies) of the included articles specified the mean HDOP for their research. For the number of satellite connections during the tracking period, 27.4% (31/113) of studies specified the mean ± SD value. This information is presented in Table 6.

**Table 5:** Tracking system characteristics

Tracking technology	Manufacturer	Device	Sample rate	Reference
Global Positioning System/Global Navigation Satellite System				
GPS	Catapult Sports	Optimeye S5	10 Hz	[6, 10, 41, 52, 78–80, 82, 83, 99, 100, 104, 117, 143]
		Optimeye G5	10 Hz	[92]
		Optimeye X4	10 Hz	[58, 61, 99, 104, 108]
		MinimaxX S5	10 Hz	[109–111, 142]
		MinimaxX S4	10 Hz	[32, 46, 47, 58, 75, 78, 80, 84–86, 103, 113, 115]
		MinimaxX S3	5 Hz	[84–86]
		MinimaxX Team Sport 2.0	5 Hz	[36–39]
		MinimaxX Team Sport 2.5	5 Hz	[66, 73, 76, 137]
			10 Hz	[50, 107, 129, 130]
		MinimaxX Team Sport 4.0	10 Hz	[40, 69, 87]
		MinimaxX	5 Hz	[64, 65, 67, 68, 144–146]
			10 Hz	[33]
	STATSports	APEX	10 Hz	[81, 82]
		Viper	10 Hz	[54, 55, 62, 71, 96–98, 105, 114, 119, 120, 122, 148]
		Viper 2	10 Hz	[43, 123, 124]
GPS	GPSports	SPI Elite	10 Hz	[17, 101, 102, 118]
		SPI HPU	15 Hz^a^	[7, 9, 11, 13, 60, 63, 94, 106, 135]
		SPI Pro	5 Hz	[90, 133, 134, 136, 137, 139, 141]
			10 Hz	[42, 132]
		SPI Pro X	15 Hz^a^	[51, 70, 74, 88, 89, 116, 127, 128, 147]
			10 Hz	[59]
	Polar	Polar Team Pro	10 Hz	[34, 72]
	Digital Simulation	SensorEverywhere	8 Hz	[49]
			16 Hz	[95, 112]
	JOHAN Sports	Johan GPS	10 Hz	[48]
	K-Sport	K-GPS	10 Hz	[35, 121]
Local positioning systems				
LPS	Catapult Sports	ClearSky T6	10 Hz	[45]
	Realtrack Systems	WIMU Pro	20 Hz	[138]
	Inmotio	Inmotio LPM	24 Hz	[125]
		Inmotio LPM	31 Hz	[126]
Radio frequency				
Radio frequency	Chyron Hego	ZXY Tracking System	40 Hz	[77]
		ZXY Tracking System	20 Hz	[56, 57, 140]
	WASP	WASP Node	10 Hz	[131]
Optical				
Optical-based tracking	ProZone Sports	ProZone 3.0	N/S	[44]
	Sport Universal Process	Amisco Pro	25 Hz	[91]
	Chyron Hego	TRACAB	N/S	[43]

^a^15-Hz device interpolated from 5 Hz

**Table 6:** GPS/GNSS data quality metrics of included studies

GPS/GNSS Data quality metric	Unit of measure	Studies that outlined variable	% of studies in review that outlined information	Reference
Horizontal Dilution of Precision (HDOP)	Mean ± SD	27/113	23.9%	[6, 9, 32, 33, 38, 40, 47, 75, 78, 81, 82, 84–86, 95, 108, 110–113, 117, 120, 129, 130, 139, 144–146]
Number of satellites connected	Mean ± SD	31/113	27.4%	[6, 9, 32, 33, 38, 47, 55, 70, 75, 80–82, 84–86, 90, 93, 95, 101, 103, 108, 110–113, 117, 120, 136, 139, 144–146]

### Acceleration Processing Characteristics

The processing methods studies implemented to calculate acceleration events are outlined in Table 7. The velocity/acceleration filters that were implemented to process athlete movement data was specified by 12.9% (16/124 studies) of the studies included in this review. The minimum effort duration for the calculation of acceleration metrics were specified in 32.3% (40/124 studies) of the included studies. The specified minimum effort duration of 0.5 s was most frequent in the included studies, followed by 0.4 s, 1 s and 0.2 s.

**Table 7:** Acceleration characteristics of included studies

Acceleration/deceleration calculation metric	Unit of measure	Minimum effort duration	Outlined in studies	% of studies in review	Reference
Velocity or acceleration filter	N/A	N/A	16/124	12.9%	[9, 32, 49, 51, 53, 63, 75, 78, 80, 91, 108, 110, 113, 125, 126, 131]
Minimum effort duration/calculation interval	Seconds (s)	0.2 s			[45, 50, 117]
		0.4 s			[36, 37, 39, 73, 80, 90, 103, 121]
		0.5 s			[32, 43, 49, 56, 57, 71, 77, 91, 95, 97, 104, 110, 120, 122–124, 126, 140, 141]
		0.6 s			[78, 108, 113]
		1 s			[70, 74, 127, 133, 145, 146]
		2 s			[65]
		**Total**	**40/124**	**32.3%**	

### Acceleration Metrics

Acceleration events in this review were quantified via numerous different metrics. These metrics encompassed counts, distance, time, load, intensity and ratio markers. Of these metrics, count-based variables were predominant. Acceleration counts were selected in 72% of the studies in this review. In total, 63% of studies included absolute acceleration counts (regardless of magnitude), whilst 32% of studies implemented acceleration counts relative to the athlete or team’s time during the activity (counts per minute). Distance (m) was next highest in terms of prevalence with 13.7% of the research in this review opting to quantify acceleration events with respect to the distance attained in threshold bands. Metrics of acceleration intensity followed, with a combined 10.9% of studies (acceleration (m s^− 2^) 6.7%, deceleration (m s^− 2^) 4.2%) opting to quantify acceleration with respect to the acceleration distance relative to the time period. Similarly, absolute acceleration was selected in 9.2% of the included studies for this review. Statistics for the acceleration metrics included are presented in Table 8.

**Table 8:** Acceleration metrics of included studies

Acceleration/deceleration metric	Unit of measure	Metric definition	% of studies featuring metric	Reference
Counts	Counts (number)	Efforts in respective threshold band	62.9%	[7, 13, 17, 34, 36–40, 42–44, 47–50, 52, 54, 55, 57, 59, 60, 62, 64–66, 69–72, 76–80, 88–90, 92, 94–99, 101–103, 105–113, 116, 117, 119, 120, 122–124, 126–128, 136–141, 144–148] [36–39, 51, 53, 56, 59, 63, 66, 69, 71, 73, 74, 84–86, 88, 89, 101–103, 108, 109, 112–115, 118, 121–124, 129, 130, 137, 138, 140]
	Counts (number) per minute	Efforts in respective threshold band with respect to activity time	31.7%	
	Counts (absolute and relative)	Overall absolute and relative count contribution to review	71.8%	
Distance	Metres	Acc/Dec distance attained in respective threshold band	13.7%	[7, 32, 33, 35, 40, 41, 45, 57, 67, 68, 79, 87, 93, 113, 125, 142, 143]
	Per minute	Distance in respective threshold band with respect to activity time and threshold	3.3%	[84–86, 121]
	Per hour	Distance attained in respective threshold band	0.8%	[46]
Acceleration	m s^− 2^	Intensity metric of any magnitude of acc over given analysis period.	6.7%	[7, 70, 78, 91, 93, 100, 131, 138]
Deceleration	m s^−2^	Intensity metric of any magnitude of dec over given analysis period.	4.2%	[7, 47, 70, 78, 100]
Acceleration Density Index	Avg Acc/Dec per 10 m; m s^−^	Average acceleration performed per 10 m of distance covered (Acceleration Load/Distance)	0.8%	[45]
Acceleration Load	Total Acc/Dec; m s^−2^	Sum of acceleration values across the analysed period Average absolute value of all acc/dec data relative to a defined period. Absolute value multiplied by defined duration to convert to load metric	0.8%	[45]
	AU		0.8%	[135]
Average Accel/Decel	(m s^−2^)	Absolute acceleration/deceleration values averaged across the specified period	9.2%	[6, 7, 9–11, 45, 58, 61, 81–83]
Time	Seconds	Time in respective threshold band	4.2%	[7, 75, 93, 104, 113]
	% time	Time spent as a percentage in respective threshold band	0.8%	[84]
	% time per minute	Percentage time spent in respective threshold band with respect to activity time and threshold	1.7%	[85, 86]
	Minutes per count	Efforts in respective threshold band with respect to activity time	2.5%	[132–134]
Ratio of Dec:Acc	Ratio Dec:Acc	Duration of Dec (High) and Dec (Mod) divided by total Acc time (High + Mod) in each period.	0.8%	[104]

## Discussion

The aim of this systematic review was to outline and compare the different methods that have been adopted to quantify acceleration events in previous team sport research. The main finding in this review was that the vast majority of included studies elected to quantify acceleration events using GPS/GNSS technology (113/124 studies) and via the use of count-based metrics (72% of all studies featured counts). Whilst the aim to ascertain how accelerations were quantified by way of metrics was achieved, this review could not achieve the secondary aim which was to determine how acceleration events were commonly processed in team sport research. Specifically, there was a lack of information provided by the studies in this review that outlined the filtering processes of acceleration events and the minimum effort duration in which these events were designated. In this review, only 13% of studies specified the filtering settings of their acceleration data whilst 32% outlined the minimum effort duration. Moreover, for GPS/GNSS research, the reporting of HDOP and the number of satellites was only specified in approximately a quarter of all eligible studies. Given the known influence of data quality metrics, filtering techniques and calculation intervals on acceleration/deceleration as it’s calculated, future team sport research should endeavour to outline how acceleration and deceleration events are processed.

### Variables Chosen to Quantify Acceleration

The results of this review overwhelmingly highlight the use of counts to outline the external acceleration load of team sport athletes. Counts and, to a lesser extent, counts relative to time accounted for the vast majority (counts 72% of all metrics) of acceleration variables selected by team sport researchers. The use of counts is not surprising given the practicality of implementing count-based metrics into the athlete monitoring process. Counts are advantageous to the practitioner for a number of reasons. Firstly, this is due to the ability to detail the number of actions occurring, usually with respect to particular thresholds. The volume of counts provides an indication of the total acceleration load and, when coupled with activity time of the athlete, can also provide an indication of the acceleration intensity. Secondly, it is relatively simple for a practitioner to apply thresholds to count metrics via the manufacturer proprietary software. This simplicity allows for efficient processing and analysis of the external acceleration load of the athlete or team.

In isolation, outlining external acceleration load via counts is an acceptable choice for most researchers and practitioners. However, counts are regularly implemented in conjunction with velocity-based thresholds that may separate efforts into corresponding bands [8]. Despite the use of threshold bands being a common practice in applied sport science, this method is limited by the validity and reliability of the athlete-tracking system recording the event [7]. Specifically, threshold-based counts for accelerations have been set at discrete intervals which may separate counts from being moderate or high with small differences separating the bands. For example, Bauer et al. [40] presented external acceleration load using count thresholds of 0–2.77 m s^− 2^ (low) and > 2.78 m s^− 2^ (high). Similarly, Blair et al. [42] specified low acceleration counts at 1.5–2.5 m s^− 2^ and high counts at > 2.5 m s^− 2^. Whilst it is logical to define a lower and upper threshold for each band, counts are also influenced by the level of error in the wearable technology device [7, 26]. For example, in Buchheit et al. [149], large inter-unit variations were found between GPS devices in acceleration and deceleration counts (coefficient of variation (CV) 10–56%) during a team sport movement simulation [7]. Following on from the research in Buchheit et al. [149], Delaney et al. [7] raised the issue that the variation seen in the aforementioned study could have been a result of the use of threshold-based counts. Specifically, the use of discrete bands for count-based acceleration events was suggested to be subject to the device reliability and that the cut-off threshold could then be subject to between-device variation. Using the example provided by Delaney et al. [7], a 3 m s^− 2^ cut-off could be measured differently by two different tracking devices. One device may measure the event at 2.98 m s^− 2^, which would not qualify for the cut-off, whilst the other may measure the effort at 3.01 m s^− 2^, which would constitute an event. It is then problematic if one device records the effort as an event, whilst the other does not, which may create inconsistencies in both the literature and the athlete monitoring process.

Issues surrounding the reliability of threshold-based variables also apply to the acceleration metrics that are measured in terms of distance (metres). Outside of the count-based metrics, distance-based acceleration variables were the third most frequent (18% combined) metric implemented by the included studies in this review. Despite sharing similar advantages to the use of count variables, distance-based metrics are also susceptible to similar issues of inter-unit reliability, particularly at moderate to high acceleration thresholds. In Thornton et al. [26], a team sport simulation circuit was implemented to identify the inter-unit reliability for three commercially available GPS/GNSS devices. For acceleration metrics, software-derived, moderate acceleration distance for STATSports APEX units were classified as having poor reliability (CV; 90% confidence limit 19.7%; ± 1.5%) whilst GPSports EVO (2.7%; ± 1.5%) and Catapult Sports S5 (3.1%; ± 1.6%) devices showed greater reliability. The substantial variation seen across the results of the three GPS/GNSS devices highlights the potential issues associated with threshold-based variables of acceleration metrics as measured by athlete-tracking devices [26]. Moreover, interchanging tracking/positional systems (e.g., GNSS & LPS) can also provide reliability issues between technologies for practitioners and researchers [150]. Given the increased use of LPS and camera-based systems within outdoor stadiums, practitioners may need to change between technologies depending on their training and competition locations [26]. Research from Buchheit et al. [150] highlighted *small to very large* variation from one LPM system (Inmotio) against GPS (GPSports SPI Pro XII & VX VX340a) and a semi-automated camera system across acceleration efforts (> 3 m s^− 2^) during match play analysis of the study. With the results of the aforementioned study, any variability between tracking systems may then have practical implications for practitioners. Generally, athletes complete the same team drills and therefore have an expectation surrounding the respective external loads associated with those drills.

A suggested way to alleviate the concerns with inter-unit variability in count-based approaches is to assign a wearable tracking device to an athlete for the duration of the competitive season [7, 151]. Whilst this suggestion is important to maintain consistency in the load reporting for each athlete, it is not without limitation. The wearable tracking device may consistently measure under the count threshold which may have practical implications for the practitioner and researcher [7]. Moreover, at the applied level, it is not uncommon to group athlete positional data together to gain an understanding for training and match loads [7]. If the combined positional average data has existing variability at the individual athlete level, this may then extend into variation seen in the group average [7]. This review anticipates the implementation of count, distance, and other threshold-based metrics in the reporting of acceleration load will continue in future team sport research. However, it is important that researchers and practitioners understand the respective limitations outlined in these metrics before choosing to incorporate them in athlete load monitoring workflows.

### Choice of Athlete Tracking System

Whilst this review sought to include all forms of athlete-tracking technology that outlined acceleration or deceleration loads, it is overwhelmingly clear that GPS/GNSS remains the most abundant and popular tracking technology within team sport research. From the results of this review, 113 out of the possible 124 studies (91%) implemented GPS or GNSS devices to track athlete locomotion. This is not surprising given these devices were largely introduced in ~ 2004 and as such have seen continued developments in their technology as well as their commercial availability to practitioners [2, 152]. The continued progressions in the capabilities of GPS/GNSS devices, with regard to improvements in device sample rates, along with the allowance to wear these devices in most major competitions, have seen these tools become commonplace in the load monitoring of team sport athletes [1, 8, 22]. The widespread acceptance of these devices (at the applied level) can be attributed to the many benefits GPS/GNSS provide the practitioner. These tools provide objective and unobtrusive data collection from the athlete on their external loads in real time, which can be further analysed to develop training programs and activity profiles aimed at preparation for competition [3] . This is aided by the nature of outdoor team sports, particularly those conducted at stadia/practice facilities with no overhanging structures or surrounding infrastructure that may occlude or partially occlude the sky. With minimal occlusion, GPS/GNSS satellite signal connection is maintained and therefore allows for improved athlete-tracking data quality. In turn, there is no additional GPS/GNSS device setup required by the practitioner, which enhances the practicality of tracking athlete movement during training and competition [2].

### Distribution of GPS/GNSS Devices

The results of this review saw the utilisation of 21 different GPS/GNSS device models from seven manufacturers in the outlining of acceleration and deceleration loads from the study cohort. Whilst the inclusion criteria of this review only included GPS/GNSS devices with sample rates at or above 5 Hz, there was a representation of both 5-Hz and 10-Hz devices from manufacturers. It is generally accepted that the use of 5-Hz GPS technology is disadvantageous compared to the greater capacities of 10-Hz devices, particularly at high-intensity acceleration and decelerations [3, 21]. In the context of the calculation of acceleration and deceleration however, the number of manufacturers and GPS/GNSS devices used, regardless of sample rate, raises concern surrounding data consistency in reporting and methodology. The concern surrounding the number of GPS/GNSS devices used stems from the known differences that exist in the data filtering methods and minimum effort durations utilised between manufacturers in the calculation of acceleration [26, 27]. This review is not suggesting that the number of devices or manufacturers of wearable technologies is an issue, but rather the issue lies in the differences in their methods to calculate acceleration. With the number of the devices and manufacturers seen in this review, it is anticipated that at least on the manufacturer level, differences exist in acceleration processing [26]. The difference in acceleration processing may then extend between device models, device firmware and between the proprietary software processing acceleration data [26]. Ultimately, variation between tracking devices could have the potential to create technology-driven rather than athlete-driven differences in acceleration/deceleration loads [26].

### Local Positioning Systems in Team Sport Research

#### Background

Historically, it has been difficult for indoor-based team sports to capture their external athlete loads during training and competition [153, 154]. Despite the continued growth of GPS/GNSS technology for outdoor team sports, the obvious limitation of enclosed stadium infrastructure means that GPS/GNSS signals cannot accurately penetrate and track indoor sports [131]. As a consequence, there has been limited technology available to indoor team sport practitioners to adequately capture external athlete loads with sports such as Basketball, Netball, Handball and Futsal relying upon optical systems to track athlete locomotion [153]. The introduction of local positioning systems (LPS) or local positioning measurement (LPM) however has seen sustained development since the inception of Radio Frequency Identification (RFID) systems [154–158]. Previously suggested to be the most abundant LPS within applied sport science, RFID systems operate by measuring the distance between anchor nodes at known locations around the field of play with athletes wearing the mobile nodes [154, 159]. Acceptable levels of accuracy exist during locomotion for RFID systems for measuring distance (mean error 1.26–3.87%) and for average and maximal velocity (3.54% and 13.15%, respectively) [155, 158, 159]. However, RFID systems can be limited by incidents of signal instability and interference [159, 160]. The developments of LPS systems that operate via Ultra-Wideband (UWB) technology have been suggested to overcome the limitations of signal instability in RFID systems [153, 159]. The enhanced technology seen in UWB systems allows for greater precision, with signals that are capable of penetrating many structural materials [153, 160]. The existing literature evaluating UWB-based LPS systems is limited but two UWB systems (WIMU Pro & Catapult ClearSky T6) are a valid means to assess the positioning of indoor court athletes [153, 154, 159, 161]. Operationally, LPS devices operate through short-range communication wave generators that are in contact with receivers [153]. Local positioning system receivers are fixed to various points around the stadium to maximise full court coverage of the technology [153].

#### Interaction of LPS Systems with Outdoor Team Sport Tracking

Whilst LPS-based studies represented a small contribution to the overall review, it is important to discuss the interaction of UWB and radiofrequency technology with outdoor team sport tracking. Given the development of UWB technology, the recent validation studies and the requirement for tracking system technology for indoor-based team sport athletes, it is anticipated that the use of LPS to measure acceleration load will continue [153]. The prevalence of UWB LPS can be seen in applied sport science with the increasing utilisation of LPS in outdoor-team sport stadia [2, 26]. With the exception of the use of optical tracking in soccer, many outdoor team sports have historically tracked external athlete loads in training and competition using GPS/GNSS technology. However, during outdoor-team sport competition in stadiums with obtrusive infrastructure, there have been instances of disruptions in signal quality. The disruptions may occur from overhanging stadium structures which disrupt the signal line of sight with satellites. To alleviate signal quality concerns, UWB LPS technology has been erected within outdoor stadia to remove the signal interference seen in GPS/GNSS data [26]. It may be that with further UWB LPS development, these systems will be preferred over the traditional GPS/GNSS devices during competition within large stadiums. Regardless, the development of LPS for indoor-based team sports is important for the analysis of the acceleration load of these athletes. However, it must be presented to practitioners that LPS technology is not without limitation. To utilise LPS, stadia must be appropriately fitted with the correct infrastructure before tracking can take place. This cost is expensive and may be problematic with venues that facilitate sporting and entertainment events [159]. Similarly, to utilise this technology for away fixtures, the LPS infrastructure must be installed in the away venue which requires compatible technology to be of use [26].

### Alternative Acceleration Metrics

The results of this review identified metrics outside of the traditional threshold-based variables for quantifying acceleration. This review identified that team sport researchers have implemented the absolute acceleration variable to quantify acceleration load. Specifically, 9% of the studies included in this review presented the absolute acceleration metric, with the majority of the studies originating from the same research group [6, 7, 10, 11, 45, 58, 61, 81–83, 135]. Absolute acceleration combines the absolute value of all acceleration data (regardless of the magnitude) and is averaged over the given time period (e.g., drill or match) [9]. The use of absolute acceleration avoids the issue of dichotomising a continuous variable into acceleration thresholds, as all acceleration events are included and are not subject to device reliability issues that are seen with threshold-based metrics [162]. For athlete load monitoring, incorporating all acceleration events may be beneficial as all acceleration events carry a physiological and mechanical cost that needs to be accounted for [7]. At the research level, the reliability of this method was also found to be *good* to *moderate* in both 5-Hz (CV 5.7%) and 10-Hz (CV 1.2 %) devices [7] when compared to VICON [163], rendering the variable suitable for team sport monitoring.

Since the introduction of the absolute acceleration metric, there have been derivative metrics of this variable introduced into research [9]. Firstly, acceleration density index (ADI) (avg Acc/Dec per 10 m; m s^− 2^) incorporates the absolute acceleration metric, but is calculated as absolute acceleration performed per 10 m of distance covered [45]. In essence, ADI is analysing acceleration load relative to distance [45]. At the applied level, ADI may provide benefit to court-based sports such as Netball or Basketball where athletes may not accumulate high acceleration load relative to total activity time (subject to rest), but accumulate substantial acceleration load during locomotion (e.g., goal shooters/goal keepers in netball or centres/power forwards in basketball) [45]. Secondly, load measures that derive from absolute acceleration were evident in this review. Acceleration total load (total Acc/Dec; m s^− 2^) summates the accumulation of all acceleration events over an analysed time period [45]. For athlete monitoring, total acceleration load can be implemented as a standalone metric or it can be used as a supplementary variable which summates the information in threshold-based acceleration metrics [45]. Similarly, acceleration load (arbitrary units; AU) featured in this review, was quantified by calculating absolute acceleration over the analysed period before multiplying the value by duration to convert to load (AU) [135]. With the growth of the absolute acceleration metric and the subsequent derivate metrics, the implementation of these variables both practically and in research is likely to continue.

### Limitations of Included Studies

With the increasing prevalence of athlete-tracking technologies in applied sport science, there has been a requirement for standardised processes when collecting and reporting upon athlete datasets [1, 8]. The basis for a standardised collecting and reporting process is to ensure greater consistency and transparency when reporting activity profiles or external athlete load in research. In keeping with the recommendations outlined by Malone et al. [1], this review attempted to extract values surrounding the quality of satellite data when tracking athletes over the analysed period. Specifically, this review analysed the HDOP and the number of satellites connected to devices during the analysed activity. The horizontal dilution of precision provides a value of the accuracy of the GPS/GNSS horizontal positional signal as determined by the geographical positioning of the satellites [164]. Generally, when satellites are spread out, HDOP is low which enhances data quality [25, 165]. To rank HDOP quality, a scale of 1–50 is implemented [1, 25]. Any HDOP value below 1 is considered optimal for HDOP readings with at least four to six satellites being required to capture human movement [1, 25]. Despite the importance of these metrics pertaining to the data quality of each individual study, this review was limited by a lack of information surrounding HDOP and the number of satellite details. For HDOP, only 24% of the eligible GPS/GNSS studies specified a HDOP value for their respective study. Similarly, only 27% of studies outlined the mean number of satellites connected to the tracking device during the analysed periods. Consequently, it is difficult to make inferences regarding the studies included in this review without sufficient information regarding their data quality. Moreover, at an applied level, it is then difficult for practitioners to make judgements regarding activity profiles. The authors do acknowledge however that whilst all GPS/GNSS devices are capable of collecting HDOP and information on the number of satellites, the access to this information may be limited by device manufacturers, which in turn may not have been made available to researchers [1]. However, with the availability of GNSS planning tools, researchers and practitioners are still be able to obtain information relating to the availability of satellites and HDOP measures during data collection. Planning tools should be consulted to document the satellite activity during the data collection to supplement the satellite information from GPS/GNSS devices. Future research should endeavour to specify HDOP and satellite information where possible to allow researchers and practitioners a wholistic opportunity to evaluate research data quality.

Despite the potential differences that may exist between athlete-tracking device hardware and specifications (e.g., device sample rate), the way in which acceleration events are calculated can result in substantial variation in acceleration load [1, 26, 27]. It is accepted that different athlete-tracking devices and manufacturers process acceleration events in different ways. Firstly, acceleration is not directly measured by the tracking device. As a result, acceleration is calculated as a derivative measure of velocity (for GNSS) [24, 166]. Secondly, there is a sweeping issue with the reporting of athlete-tracking data in which there is no consensus method to process acceleration events. These two points coupled with the increasing amount of wearable tracking devices and manufacturers available to practitioners has potentially created technology-driven variations in acceleration load between devices [1, 26]. Variations include the filtering of velocity and/or acceleration data by device manufacturers and also the selection of minimum effort durations (MED) for acceleration events [8, 27].

The filtering of athlete tracking data can directly influence acceleration load, regardless of the magnitude or metric used to quantify the event [1, 8, 26, 27]. The purpose of filtering extends to maintaining data quality, removing poor signals and to decreasing the noise content of the signal [23, 166–169]. In human movement, there are many different types of filters which have been introduced to process athlete data [23]. Firstly, bandpass filters help to convert raw data from the spatial to the time domain via the use of a Fourier Fast Transform (FFT) [23, 166, 170]. The use of low pass filters allows for low-frequency signals to pass whilst minimising the high-frequency noise, whereas digital filtering processes the frequency spectrum of the noise and the signal [23]. In LPS, common filtering methods include, but are not limited to, Kalman and Butterworth filters, whilst GPS/GNSS devices can also utilise Butterworth as well as moving average, moving median, median or exponential filters [1, 23, 49, 63, 131, 157, 158, 166]. However, the process by which manufacturers select their filtering process is arbitrary and can vary from manufacturer to manufacturer [1]. In research and for applied sport science practitioners, this is problematic as there are many different manufacturers and devices commercially available. As such, there are many different types of filters that can be modified, potentially altering the magnitude of an acceleration event [26]. For example, manufacturers may elect to filter the velocity trace using a determined filter and then calculate acceleration from the velocity trace. Manufacturers may also filter the velocity trace and then filter the calculation of acceleration using a predefined filter. Therefore, consistency in the reporting of filtering methods is required when processing athlete acceleration data. In this review, only 13% of studies detailed the filter used when processing athlete movement data. This detail includes proprietary filters as defined by the manufacturers and custom filters applied by researchers. The lack of information surrounding the filtering processes in these studies then raises questions as to any identified differences between the research. Are these differences driven by the discrepancies between athlete-based external outputs or are they derived from technology-driven influences from the use of different data processing methods [26]? However, in posing this question, the researchers do acknowledge that in similar regard to satellite and HDOP information, the filtering process used in the calculation of acceleration via the manufacturer’s proprietary software may not be made available.

With the lack of critical information on filtering and signal quality, the authors of this review were limited in the ability to make judgements and comparisons on acceleration. It is difficult to assess external athlete load without knowing how the acceleration data were processed, given the known influence these processes have on athlete external loads [1, 8, 26]. Therefore, it is important that future research outlines the filtering processes used in the calculation of acceleration to ensure appropriate comparisons between tracking technology and external athlete load. However, if future research begins to improve the reporting process on filtering in the calculation of acceleration, there may still be issues surrounding the comparability of acceleration load between athlete-tracking technologies and manufacturers. There may still be technology-driven discrepancies between activity profiles and validity and reliability studies of wearable technology [26]. Following the summations of Thornton et al. [26], this review contends that future research should be centred towards a consistent method to process acceleration. Despite the majority of the discussion surrounding GPS/GNSS technology, it is anticipated that these same difficulties would occur with local positioning systems and optical systems [26].

The minimum effort duration (MED) is a qualifying criterion in which acceleration events need to be sustained for a specific time frame for the effort to be acknowledged as an event [8, 27]. For instance, if a MED of 0.5 s was chosen, the athlete would need to maintain the acceleration for at least 0.5 s for it to qualify as an event [27]. However, the selection of the MED is problematic as the MED and any accompanying velocity threshold (where applicable) is generally arbitrary. The arbitrary selection of the MED may be due to many factors including the inconsistency in the selection of the MEDs within previous team sport research and the use of different tracking devices and manufacturers. Currently, there is no consensus or consistent MED outlined in athlete-tracking-based studies and as such, there has been a wide variety of different MEDs presented to calculate external athlete acceleration load [8, 27]. In this review, there were six different MEDs selected, ranging from 0.2 s to 2 s, with the 0.5 s threshold being the most frequent. Moreover, approximately 68% of the included studies in this review did not specify their MED for acceleration or deceleration events.

The variation in MEDs between studies is in itself problematic, as the calculation interval directly influences the magnitude of an acceleration [8, 27]. In the Harper et al. [8] review, the study made the point that small fluctuations between MED intervals (i.e., 0.1 s) can result in differences in the number of high-intensity acceleration efforts. The suggestion from Harper et al. [8] is based on the original research from Varley et al. [27], which quantified the impact of differing MEDs (from 0.1 s to 1.0 s (0.1 s increments)) upon acceleration counts. In this research, the authors concluded that during an elite Dutch soccer match, there was an exponential decline in the number of observed acceleration efforts as the MED increased, across all filtering methods [27]. In essence, this finding confirmed that the selection of a lower MED of 0.1–0.3 s (GNSS) is more appropriate for capturing short and discrete acceleration events [8]. However, MEDs of 0.1-0.3 s (GNSS) in length are also more susceptible to any error in measurement that may be a result of numerous repeat accelerations that occur too closely together [8]. Conversely, a MED of longer duration (> 0.5 s) may have a smoothing effect on the acceleration datapoints for GNSS-based technology, which in turn may dampen the magnitude of higher acceleration events or may underestimate the number of efforts [27]. It should be stressed that this research is GNSS based and may have different implications for LPS/LPM technology.

There is no one “perfect” MED for the calculation of athlete acceleration [27]. However, it is prudent for practitioners to realise the implications of the selection of a MED and how this may be compared with similar team sport activity profiles [27]. It is also recognised by the researchers that the choice of a MED may be dictated by the tracking device model/manufacturer. Similar to the choice of filtering applied to acceleration data, practitioners may be limited to the MED specifications outlined by the manufacturer, whilst other manufacturers may allow complete customisation of the process. Regardless of the situation, differences in MED settings can still lead to differences in acceleration load between research studies.

To alleviate the potential differences in load as a result of different MED settings, previous research has highlighted the use of a threshold inclusion criteria [8, 27]. The inclusion criteria suggested that a qualifying threshold standard for an acceleration effort could be implemented alongside a MED. For example, the acceleration must eclipse 1 m s^− 2^ for the effort to be counted. Moreover, to establish an acceleration endpoint for an effort, this could be implemented when acceleration falls below 0 m s^− 2^ [8, 27]. The issue of varied MEDs in research however still exists with this method. With inconsistencies seen between MEDs in this review, future research may then look to identify appropriate MEDs with respect to each team sport. The presence of MEDs with respect to each team sport would then create a more consistent approach to acceleration/deceleration reporting.

### Future Research

To improve future research, studies should attempt to improve the consistency in the processing and reporting of team sport acceleration and deceleration loads. Specifically, future research should be guided by the following recommendations:

- Report the HDOP and number of satellites in connection with devices during data collection (satellite-based technology only).
- Report the acceleration processing method, including any filtering methods (if known and applicable) and the minimum effort duration.
- Utilise GNSS planning tools (where applicable) to evaluate the performance of their respective wearable tracking system relative to the available satellites (satellite-based technology only).
- Move towards the determination of a common acceleration filter that can be used practically and within research that may be sport specific.

When reporting acceleration load from tracking devices, it is important that future studies attempt to outline the HDOP and the average number of satellites in connection with the devices during analysis. Satellite information can be used by researchers and practitioners as an indication of the signal quality from these devices and can aid in the evaluation of the quality of the acceleration/deceleration datasets. In terms of acceleration metrics, future research should also endeavour to outline the acceleration filtering used to process the acceleration data (if known and applicable) and the MED to quantify any threshold-based metrics.

Future research should attempt to introduce a common acceleration filtering technique for the processing of external athlete acceleration and deceleration loads. A common filtering technique that is sport specific may be appropriate. However, the amount of tracking devices, manufacturers and systems seen in this review highlights the importance of having a consistent process to handle and process acceleration data. Without a consistent process and with the known influence filtering methods have upon acceleration/deceleration data, future research will continue to question whether differences in acceleration/deceleration loads are athlete or technology driven [26].

## Conclusions

Acceleration metrics are important components of the external load monitoring process of team sport athletes. The ability to quantify acceleration events allows practitioners to understand the energetic (acceleration-focused) and eccentric load placed upon the athlete during training and competition [7]. With athlete acceleration information, acceleration-specific loads can be accounted for in the athlete preparatory process.

Acceleration events in team sport research have been predominately quantified via the use of effort counts, including counts related to time. Other “traditional” metrics in terms of acceleration being quantified via distance remains a relevant selection, as does average intensity by practitioners.

Global Positioning Systems and now GNSS are the most common tracking systems utilised in the quantification of acceleration in the team sport athlete. However, despite the widespread use of GPS/GNSS technology in tracking athlete locomotion, there is a lack of information surrounding the signal quality via the HDOP and number of satellite metrics. Future research should aim to outline HDOP and the number of satellites where possible, to allow researchers to evaluate the quality of the athlete tracking data.

The calculation of acceleration in the athlete-tracking device is influenced by MEDs and the specification of data filtering processes. Despite the influence and variation of data filtering and MEDs between tracking device manufacturers, these metrics have not been consistently published in research. This review concludes that even if future studies outlined the acceleration data filtering process, the anticipated variation between tracking manufacturers and devices may highlight technology-driven influences in acceleration/deceleration loads. Therefore, a consistent and potentially sport-specific acceleration filtering process and reporting structure needs to be developed and introduced within applied team sport research.

## Data Availability

Not applicable.

## Abbreviations

AU: Arbitrary units
Acc: Acceleration
ADI: Acceleration density index
CV: Coefficient of variation
Dec: Deceleration
FFT: Fast Fourier Transform
GNSS: Global Navigation Satellite System
GPS: Global Positioning System
HDOP: Horizontal Dilution of Precision
LPM: Local Positioning Measurement
LPS: Local Positioning System
MED: Minimum effort duration
PRISMA: Preferred items for Systematic Reviews and Meta-Analyses
RFID: Radio Frequency Identification systems
UWB: Ultra-wideband
